# Epilepsy Detection in EEG Using Grassmann Discriminant Analysis Method

**DOI:** 10.1155/2020/2598140

**Published:** 2020-05-01

**Authors:** Hongbin Yu, Chao Fan, Yunting Zhang

**Affiliations:** ^1^School of Artificial Intelligence and Computer Science, Jiangnan University, Wuxi 214000, China; ^2^Institute of Commerce of Digital Art Animation, Wuxi 214000, China

## Abstract

Epilepsy is marked by seizures stemming from abnormal electrical activity in the brain, causing involuntary movement or behavior. Many scientists have been working hard to explore the cause of epilepsy and seek the prevention and treatment. In the field of machine learning, epileptic diagnosis based on EEG signal has been a very hot research topic; many methods have been proposed, and considerable progress has been achieved. However, resorting the epileptic diagnosis techniques based on EEG to the reality applications still faces many challenges. Low signal-to-noise ratio (SNR) is one of the most important methodological challenges for EEG data collection and analysis. This paper discusses an automated diagnostic method for epileptic detection using a Fréchet Mean embedded in the Grassmann manifold analysis. Fréchet mean-based Grassmann discriminant analysis (FMGDA) algorithm to implement the EEG data dimensionality reduction and clustering task. The method is resorted to reduce Grassmann data from high-dimensional data to a relative lower-dimensional data and maximize between-class distance and minimize within-class distance simultaneously. Every EEG feature is mapped into the Grassmann manifold space first and then resort the Fréchet mean to represent the clustering center to carry out the clustering work. We designed a detailed experimental scheme to test the performance of our proposed algorithm; the test is assessed on several benchmark datasets. Experimental results have delivered that our approach leads to a significant improvement over state-of-the-art Grassmann manifold methods.

## 1. Introduction

Epilepsy is a critical neurological disease that is caused by temporary abnormal discharges of the brain electrical activity, leading to uncontrollable movements and tremulous [1]. People with epilepsy are two or three times more likely to die early than the normal person [2]. It was estimated that approximately 1% people in the world suffer from epilepsy, and elderly patients are the majority [3]. Hence, epilepsy detection is of great significance in clinical therapy of epileptic patients.

Electroencephalogram (EEG) is most commonly used in epilepsy detection manner, since it contains valuable physiological information about the brain; it is also a valuable clinical tool for epilepsy evaluation and treatment [4]. Many automated diagnostic systems for epilepsy have been developed based on different technologies. In the area of artificial intelligence, many researchers have been focusing on the research of EEG data dimensionality reduction and discriminant feature extracting from the raw EEG data as well as exploring high-performance clustering model to implement the epilepsy detection task.

Many feature extraction methods have been developed for the epilepsy detection including time-domain [5, 6], frequency-domain [7–12], time-frequency analysis [13], energy distribution in the time-frequency plane [14], wavelet features [15], and chaotic features such as entropies [6, 16]. There are still many classification methods that have been proposed to solve the epilepsy detection problems in recent years. Dehuri et al. proposed to use DE-RBFN method to implement the epilepsy detection task; EEG signals are decomposed with wavelet transform into different subbands, and statistical information is extracted from the wavelet coefficients to supply as the input to ensemble of DE-RBFNs [1]. Li et al. proposed to use the discrete wavelet transform (DWT) in combination with EA method to extract significant features from raw EEG signals, and an effective network model called NNE is designed specifically to the task of epilepsy detection [2]. Jiang et al. propose to use multiple feature extraction method to obtain the multiview feature from the raw EEG, and the classical Takagi-Sugeno-Kang fuzzy system is used to implement the classification task [17]. Zhang et al. extracted the entropy as the feature and combine the SVM classifier to estimate the epileptic cases based on the EEG signal [18].

However, the aforementioned algorithms for epilepsy detection based on EEG data are all concerned in terms of Euclidean alike distance. In order to extend the study of EEG signal to non-Euclidean space and solve these problems existing in the EEG applications that the traditional Euclidean algorithms cannot solve, in this paper, we will cast the EEG processing problem into the Grassmann manifold to implement the epilepsy detection task.

Grassmann manifold have been a popular method in recent years for its strong capability in extracting discriminative information for image sets and videos, where the datasets are vectors instead of the normal used real value vectors. For instance, in the study of computer visions, except the traditional well-structured data, such as image data as well as video data, there exist some manifold-valued data. The movement of scattered key points in the video can be described by subspaces, i.e., the points on the so-called Grassmann manifold and the covariance feature descriptors of images are SPD manifold-valued data [19–21]. Thus, the clustering problem is completely different from those which instances are clustered into clusters according to their space stucture in the euclidean spaces. However, in the Grassmann manifold space, the objects to be clustered themselves are subspaces (of the same dimension), i.e., the points on the abstract Grassmann manifold [22] which is completely different from the traditional Euclidean space with data vector form. A Grassmann manifold *𝒢*(*p*, *D*) is the space of all *p*-dimensional linear subspaces of **R**^*D*^(0 ≤ *p* ≤ *D*). For *p* = 0, the Grassmann manifold becomes the Euclidean space itself. As Grassmann manifold is abstract, there are a number of ways to realize it, such as full column rank matrix representation method [19], orthogonal representation [23], symmetric idempotent matrix representation [24], and Stiefel manifold representation method [23]. Through those methods, some existing algorithms which are developed in the Euclidean space can be extended to the Grassmann manifold. Hamm and Lee [25] employed the projection metric to encode the Grassmannian points by Grassmannian kernels and developed Grassmann discriminant analysis on the kernel space. Further, Hamm and Lee [26] theoretically studied the relationship between projection kernel and the KL distance. Harandi et al. [27] proposed a graph embedding-based discriminant analysis approach on Grassmannian manifold which aims to simultaneously maximize discriminant power and preserve the geometrical structure of the manifold. Huang et al. [28] employed the projection metric to learn discriminant transformation on the Grassmann manifold. Wang et al. [21] extended classical LPP from the Euclidean space to Grassmann manifold. We adopt the Stiefel manifold representation strategy for the learning task in the Grassmann manifold space and proposed a Fréchet mean-based Grassmann discriminant analysis (FMGDA) algorithm for image sets recognition which has been presented in [29]. Comparing with the previously proposed Grassmann manifold algorithms, our proposed Fréchet mean algorithm has two important innovations: firstly, we solve the feature extraction problems by minimizing the within-class mean and maximizing the between-class mean simultaneously for Grassmannian points. Secondly, our proposed method generalized the classic LDA to non-Euclidean Grassmann manifolds, and our optimization problem can be characterized by the trace ratio problem.

EEG signal is an effective tool to study the firing mechanism of cortical neurons; however, due to intrinsic nature of lower SNR ratio, applications based on EEG signal are severely limited. To solve these problems that cannot be solved in the ordinary Euclidean space, we proposed to map the EEG features to the Grassmann manifold to find some clues to solve those problems and enhance the epilepsy diagnosis accuracy simultaneously. Most of the previously used algorithms for epilepsy detection are carried out in the Euclidean space, in order to extend the scope of EEG research and provide more ideas to solve the problems of epilepsy detection based on EEG signal; we proposed to implement the epilepsy detection task in the Grassmann manifold to do some exploratory research work.

Inspired by the already existing video classification research work in the Grassmann manifold space, we transform the ordinary epilepsy detection task into an image classification task, where the energy distribution in the time-frequency plane of EEG signal epochs is viewed as an image; thus, the epilepsy detection based on the EEG can be realized by implementing an image classification task. We adopt the FMGDA method to implement the epilepsy detection task. The spectrum features of each epoch EEG time series are extracted by short-time Fourier transform (STFT) first and get a spectrum matrix. The matrix is then used to construct a dataset vector, just as the video frames. The FMGDA algorithm finally carries out to classify each dataset.

We summarize our contributions as follows:

- The energy distribution in the time-frequency plane of EEG signal can be used as the image for EEG classification
- EEG classification problem can be transformed to an image clustering problem in the Grassmann manifold space
- The Fréchret mean of EEG energy distribution in the time-frequency plane can be used to characterize the clustering center in the Grassmann manifold space

The paper is organized as follows: firstly, we review the traditional linear discriminant analysis method in Section 2. In Section 3, we introduce our Fréchet mean-based Grassmann discriminant analysis (FMGDA) method. In Section 4, we evaluate our proposed algorithms' performance on the epilepsy detection. In Section 5, we conclude our work.

## 2. Fréchet Mean-Based Grassmann Discriminant Analysis

In this section, we will introduce our Fréchet mean-based Grassmann discriminant analysis (FMGDA) algorithm which was proposed in [29]. We will use our FMGDA algorithm to implement the epilepsy detection task in the subsequent section.

### 2.1. Linear Discriminant Analysis

Linear discriminant analysis has been proposed for several years and gained considerable attentions for its superiority on the dimensionality reduction, feature extraction, and the classification research.

Given *n* samples *x*_1_, *x*_2_, *x*_3_, ⋯, *x*_*n*_ from *c* classes. LDA is to find a linear transformation which can maximize the between-class distance and minimize the within-class distance simultaneously in the transformed subspace. In other words, the linear mapping *a* can be obtained by solving the following optimization problem.

(1) $$\begin{array}{c} \hat{a}=\text{argmax}\frac{a^{T}S_{b}a}{a^{T}S_{w}a}, \end{array}$$

where

(2) $$\begin{array}{c} \begin{array}{c} S_{b}=\overset{c}{\underset{k=1}{\sum }}n_{k}\left(\mu^{\left(k\right)}-\mu \right){\left(\mu^{\left(k\right)}-\mu \right)}^{T},\\ S_{w}=\overset{c}{\underset{k=1}{\sum }}\overset{n_{k}}{\underset{i=1}{\sum }}n_{k}\left(x_{i}^{\left(k\right)}-\mu^{\left(k\right)}\right){\left(x_{i}^{\left(k\right)}-\mu^{\left(k\right)}\right)}^{T}. \end{array} \end{array}$$

Here, *μ*^(*k*)^ denotes the mean vector of the *k*th class, and *μ* denotes the centroid of all the sample instances. *x*_*i*_^(*k*)^ is the *i*th instance from the *k*th class, and *n*_*k*_ denotes the instance number of the *k*th class. Matrices *S*_*b*_ and *S*_*w*_ are often called the between-class scatter matrix and within-class scatter matrix, respectively.

Substituting the sample instances into the formula, with some simple algebra manipulations, we can obtain the between-class scatter matrix and within-class scatter matrix as follows:

(3) $$\begin{array}{c} a^{T}S_{b}a=\overset{c}{\underset{k=1}{\sum }}n_{k}a^{T}\left(\mu^{\left(k\right)}-\mu \right){\left(\mu^{\left(k\right)}-\mu \right)}^{T}a,=\overset{c}{\underset{k=1}{\sum }}n_{k}{\left\|a^{T}\left.\mu^{\left(k\right)}-a^{T}\mu \right)\right\|}_{2}^{2},=\overset{c}{\underset{k=1}{\sum }}n_{k}\delta_{E}\left(a^{T}\mu^{\left(k\right)},a^{T}\mu \right),\\ a^{T}S_{w}a=\overset{c}{\underset{k=1}{\sum }}\overset{n_{k}}{\underset{i=1}{\sum }}n_{k}{\left\|a^{T}\left(x_{i}^{\left(k\right)}-\mu^{\left(k\right)}\right)\right\|}_{2}^{2},=\overset{c}{\underset{k=1}{\sum }}\overset{n_{k}}{\underset{i=1}{\sum }}n_{k}{\left\|a^{T}x_{i}^{\left(k\right)}-a^{T}\mu^{\left(k\right)}\right\|}_{2}^{2},=\overset{c}{\underset{k=1}{\sum }}\overset{n_{k}}{\underset{i=1}{\sum }}n_{k}\delta_{E}\left(a^{T}x_{i}^{\left(k\right)},a^{T}\mu^{\left(k\right)}\right), \end{array}$$

where the notation *δ*_*E*_(·, ·) denotes the Euclidean distance between two regular data vectors. The solution of (1) is the generalized eigenvectors corresponding to the largest eigenvalues of the following:

(4) $$\begin{array}{c} S_{b}a=\lambda S_{t}a. \end{array}$$

Thus, mapping vector *a* can be obtained by adopting the eigen-decomposition method on the matrix *S*_*t*_^−1^*S*_*b*_, if *S*_*t*_ is nonsingular. There are at most *c*‐1 eigenvectors corresponding to nonzero values, since the rank of *S*_*b*_ is bounded from above by *c*‐1. Thus, the reduced dimension by LDA is at most *c*‐1.

### 2.2. Grassmann Manifold

In this section, we provide a brief summary of the basic Riemannian geometry of Grassmann manifold. More details can be found in [23, 30, 31]. A Grassmann manifold *𝒢*(*p*, *D*) is the space of all *p*-dimensional linear subspaces of ℝ^*D*^(0 ≤ *p* ≤ *D*). When *p* = 0, the Grassmann manifold becomes the Euclidean space itself. When *p* = 1, the Grassmann manifold consists of all the lines passing through the origin in ℝ^*d*^. As Grassmann manifold is abstract, there are a number of ways to realize it for numerical learning purpose.

Assuming that ℝ_∗_^*d*×*p*^ be the space of all *d* × *p* matrix of full column rank, GL(*p*) denote the general group of nonsingular matrices of order *p* and *𝒪*(*p*) the group of all the *ptimesp* orthogonal matrices.

(i) Representation by full column rank matrices [19]
  (5) $$\begin{array}{c} G\left(p,d\right)≅{\mathbb{R}}_{*}^{d\times p}/\text{GL}\left(p\right) \end{array}$$
(ii) The orthogonal representation [23]:
  (6) $$\begin{array}{c} G\left(p,d\right)≅O\left(d\right)/O\left(p\right)\times O\left(d-p\right) \end{array}$$
(iii) Sysmmetric idempotent matrix representation [24]:
  (7) $$\begin{array}{c} G\left(p,d\right)≅\left\{P\in {\mathbb{R}}^{d\times d}:P^{T}=P,P^{2}=P,\mathrm{rank}\left(P\right)=p\right\}. \end{array}$$
(iv) The Stiefel manifold representation [25];
  (8) $$\begin{array}{c} G\left(p,d\right)≅ST\left(p,d\right)/O\left(p\right) \end{array}$$
  where *𝒮𝒯*(*p*, *d*) = {*X* ∈ ℝ^*d*×*p*^ : *X*^*T*^*X* = *I*_*p*_}.

In our proposed FMGDA, we adopt the Stiefel manifold representation strategy to complete our Grassmann manifold-based algorithm. A point *X* on the Grassmann manifold *𝒢*(*p*, *D*) is a subspace spanned by the orthonormal columns of a *D* × *p* matrix *X* such that *X*^*T*^*X* = *I*_*p*_, where *I*_*p*_ is the identity matrix of size *p* × *p*.

Grassmann manifold has a nice property that it can be embedded into a space consisting of symmetric positive semidefinite matrices. More precisely, let *X* ∈ *𝒢*(*p*, *D*), we can define the following projection embedding

(9) $$\begin{array}{c} \Pi :G\left(p,D\right)⟶\text{Sy}{\text{m}}_{+}\left(D\right),\Pi \left(X\right)=XX^{T}, \end{array}$$

where Sym_+_(*D*) denotes the space of *D* × *D* symmetric positive semidefinite matrices. Since Sym_+_(*D*) can be understood as a Euclidean space, a natural metric for Sym_+_(*D*) is the Frobenius norm. As such, we can define the following projection metric [25]:

(10) $$\begin{array}{c} \delta_{P}\left(X_{1},X_{2}\right)=\frac{1}{\sqrt{2}}{\left\|\Pi \left(X_{1}\right)-\Pi \left(X_{2}\right)\right\|}_{F}^{2}, \end{array}$$

where *X*_1_ and *X*_2_ are two Grassmann points and *Π*(*X*_*i*_) = *X*_*i*_*X*_*i*_^*T*^, *i* = 1, 2. As pointed in [32], the projection metric *δ*_*P*_(·, ·) is able to approximate the true Grassmannian geodesic distance and become one of the most popular metrics for analyzing Grassmann manifold features [25, 27, 33].

## 3. FMGDA

In this section, we propose FMGDA, a supervised subspace learning for Grassmann manifold that maps a high-dimensional Grassmann point to a lower-dimensional Grassmann manifold.

Suppose we have a data set (*𝒳*, *Y*), where *𝒳* = {*X*_*i*_}_*i*=1_^*n*^, *X*_*i*_ ∈ *𝒢*(*p*, *D*) is a Grassmann point, *Y* = [*y*_1_, *y*_2_, ⋯, *y*_*n*_] is the class indicator matrix. *y*_*i*_(*j*) = 1 if *X*_*i*_ belongs to the *j*th class and 0 otherwise. Our purpose is to learn the parameter *A* ∈ **R**^*D*×*d*^ of a mapping in the form *f*: *X*_*i*_ ∈ *𝒢*(*p*, *D*)⟶*𝒢*(*p*, *d*), which is defined as:

(11) $$\begin{array}{c} f\left(X_{i},A\right)=A^{T}X_{i}. \end{array}$$

With this mapping *f*, the original high-dimensional Grassmann manifold can be transformed into a lower-dimensional Grassmann manifold. However, *A*^*T*^*X*_*i*_ is not a valid Grassmann point since the parameter *A* is not an orthogonal matrix. To solve this problem, we temporarily employ the orthonormal components of *A*^*T*^*X*_*i*_ defined by *A*^*T*^*X*_*i*_′ to represent an orthonormal basis matrix, the transformed projection matrix. We now rewrite *f*(*X*_*i*_, *A*) = *A*^*T*^*X*_*i*_′ to make *f*(*X*_*i*_, *A*) a valid Grassmann point. The approach to get *A*^*T*^*X*_*i*_′ will be thoroughly discussed in Section 4.

Let *X*^(*k*)^ = [*X*_1_^(*k*)^, *X*_2_^(*k*)^, ⋯, *X*_*n*_*k*__^(*k*)^] be a set of Grassmann points from the *k*th class. Recalling the definition of LDA, we require the mean to capture the discriminant information. However, traditional Euclidean mean is not a valid Grassmann point. Therefore, special care must be taken into account to compute the subspace mean for Grassmann manifold. Fortunately, subspace mean on the Grassmann manifold has been studied in [34–36]. In particular, the Fr*é*chet mean is commonly used to characterize the subspace mean of Grassmann manifold.

**Definition 1 .**

The Fr*é*chet mean *M*^∗^ for a set of points {*X*_*i*_}_*i*=1_^*n*^, *X*_*i*_ ∈ *𝒢*(*p*, *D*) is the local minimizer of the cost function

(12) $$\begin{array}{c} M^{*}=\arg\underset{M}{\min}\overset{n}{\underset{i=1}{\sum }}\delta_{P}\left(X_{i},M\right). \end{array}$$

The above definition shows that the subspace mean depends heavily on the metric. If we assume all points come from Euclidean space and choose the Euclidean metric, the Fr*é*chet mean has a closed form solution which is nothing but the traditional mean. Unfortunately, there is usually no closed solution for *M*^∗^ with Riemannian metric, and the first-order gradient descent method [22] is commonly employed to find the solution. For Grassmann data points endowed with the projection metric, we have an analytic solution for the Fr*é*chet mean which is characterized in the following lemma.

**Lemma 1 .**

The Fr*é*chet mean *M*^∗^ for a set of points {*X*_*i*_}_*i*=1_^*n*^, *X*_*i*_ ∈ *𝒢*(*p*, *D*) is the *p* largest eigenvectors of ∑_*i*=1_^*n*^*X*_*i*_*X*_*i*_^*T*^.

Let *M*^(*k*)^ be the class Fr*é*chet mean of the *k*th samples *X*^(*k*)^ and *M* be the total Fr*é*chet mean of *𝒳*. Similar to LDA, the within-class distance and between-class distance in the transformed low-dimensional Grassmann manifold are defined as

(13) $$\begin{array}{c} \begin{array}{c} d_{w}\left(A\right)=\overset{K}{\underset{k=1}{\sum }}\overset{n_{k}}{\underset{i=1}{\sum }}n_{k}\delta_{P}\left(f\left(X_{i}^{\left(k\right)},A\right),f\left(M^{\left(k\right)},A\right)\right),\\ d_{b}\left(A\right)=\overset{K}{\underset{k=1}{\sum }}n_{k}\delta_{P}\left(f\left(M^{\left(k\right)},A\right),f\left(M,A\right)\right), \end{array} \end{array}$$

where *f*(*X*_*i*_^(*k*)^, *A*) = *A*^*T*^*X*_*i*_′, *f*(*M*, *A*) = *A*^*T*^*M*′, and *f*(*M*^(*k*)^, *A*) = *A*^*T*^*M*^′(*k*)^. Note that *A*^*T*^*M*′ and *A*^*T*^*M*^′(*k*)^ are the orthonormal components of *A*^*T*^*M* and *A*^*T*^*M*^(*k*)^, respectively. By the definition of *δ*_*P*_(·, ·), we can explicitly write *d*_*w*_(*A*) and *d*_*b*_(*A*) as follows:

(14) $$\begin{array}{c} \begin{array}{c} d_{w}\left(A\right)=\overset{K}{\underset{k=1}{\sum }}\overset{n_{k}}{\underset{i=1}{\sum }}n_{k}{\left\|A^{T}X_{i}^{\prime \left(k\right)}X_{i}^{\prime \left(k\right)T}A-A^{T}M^{\prime \left(k\right)}M^{\prime \left(k\right)T}A\right\|}_{F}^{2},\\ d_{b}\left(A\right)=\overset{K}{\underset{k=1}{\sum }}n_{k}{\left\|A^{T}M^{\prime \left(k\right)}{M^{\prime \left(k\right)}}^{T}A-A^{T}M^{\prime }M^{\prime T}A\right\|}_{F}^{2}. \end{array} \end{array}$$

It should be pointed out that *A*^*T*^*M*^′(*k*)^ does not exactly express the Fr*é*chet mean of *X*^(*k*)^ in the lower dimensional manifold, but expresses it only approximately. Following the idea of LDA, we now arrive at the objective of FMGDA

(15) $$\begin{array}{c} \underset{A}{\max}\frac{d_{b}\left(A\right)}{d_{w}\left(A\right)}, \end{array}$$

which aims to maximize the between-class distance and minimize the within-class distance simultaneously.

## 4. Iterative Optimization

The optimization problem (15) includes four variables *A*, *X*_*i*_^′(*k*)^, *M*′ and *M*^′(*k*)^ which is hard to find a closed solution. In the following, we propose an iterative solution for one of the four variables at a time by fixing the other and repeating for a certain number of iterations.

We follow the work in [21, 28] to obtain the orthonormal components of *A*^*T*^*X*_*i*_^′(*k*)^, *A*^*T*^*M*′, and *A*^*T*^*M*^′(*k*)^. Specifically, let *A*^*T*^*X*_*i*_^(*k*)^ = *Q*_*x*_*i*__*R*_*x*_*i*__ be the QR decomposition, where *Q*_*x*_*i*__ ∈ **R**^*d*×*p*^ is an orthogonal matrix and *R*_*x*_*i*__ ∈ **R**^*p*×*p*^ is a nonsingular upper-triangular matrix. It is easy to show that *Q*_*x*_*i*__ = *A*^*T*^*X*_*i*_^′(*k*)^, where *X*_*i*_^′(*k*)^ = *X*_*i*_^(*k*)^*R*_*x*_*i*__^−1^. Note that *Q*_*x*_*i*__ and *A*^*T*^*X*_*i*_^(*k*)^ represent the same subspace and *Q*_*x*_*i*__ is orthonormal. Similar normalization procedure can be applied to get the other two variables *M*′ and *M*^′(*k*)^.

Let *B*^(*k*)^ = *M*^′(*k*)^*M*^′(*k*)^^*T*^ − *M*′*M*^′*T*^, we have

(16) $$\begin{array}{c} d_{b}\left(A\right)=\overset{K}{\underset{k=1}{\sum }}n_{k}{\left\|A^{T}M^{\prime \left(k\right)}{M^{\prime \left(k\right)}}^{T}A-A^{T}M^{\prime }M^{\prime T}A\right\|}_{F}^{2},=\overset{K}{\underset{k=1}{\sum }}n_{k}{\left\|A^{T}B^{\left(k\right)}A\right\|}_{F}^{2},=\overset{K}{\underset{k=1}{\sum }}n_{k}\text{tr}\left(A^{T}B^{\left(k\right)}AA^{T}B^{\left(k\right)}A\right). \end{array}$$

Similarly, let *Q*_*ik*_ = *X*_*i*_^′(*k*)^*X*_*i*_^′(*k*)^^*T*^ − *M*^′(*k*)^*M*^′(*k*)^^*T*^, we have

(17) $$\begin{array}{c} d_{w}\left(A\right)={\left\|A^{T}X_{i}^{\prime \left(k\right)}{X_{i}^{\prime \left(k\right)}}^{T}A-A^{T}M^{\prime \left(k\right)}{M^{\prime \left(k\right)}}^{T}A\right\|}_{F}^{2},=\overset{K}{\underset{k=1}{\sum }}\overset{n_{k}}{\underset{i=1}{\sum }}n_{k}{\left\|A^{T}Q_{ik}A\right\|}_{F}^{2},=\overset{K}{\underset{k=1}{\sum }}\overset{n_{k}}{\underset{i=1}{\sum }}n_{k}\text{tr}\left(A^{T}Q_{ik}AA^{T}Q_{ik}A\right). \end{array}$$

We now define a new objective *g*_*t*_(*A*) in the *t*th iteration by using the last step *A*^(*t* − 1)^ as follows

(18) $$\begin{array}{c} g_{t}\left(A\right)=\frac{tr\left(A^{T}{\tilde{B}}^{\left(t-1\right)}A\right)}{tr\left(A^{T}{\tilde{Q}}^{\left(t-1\right)}A\right)}, \end{array}$$

where

(19) $$\begin{array}{c} {\tilde{B}}^{\left(t-1\right)}=\overset{K}{\underset{k=1}{\sum }}n_{k}B^{\left(k\right)}A^{\left(t-1\right)}{A^{\left(t-1\right)}}^{T}B^{\left(k\right)},\\ {\tilde{Q}}^{\left(t-1\right)}=\overset{K}{\underset{k=1}{\sum }}\overset{n_{k}}{\underset{i=1}{\sum }}n_{k}Q_{ik}A^{\left(t-1\right)}{A^{\left(t-1\right)}}^{T}Q_{ik}. \end{array}$$

Clearly, the solution of *t*th iteration can be stated as

(20) $$\begin{array}{c} \underset{A}{\max}\frac{\text{tr}\left(A^{T}{\tilde{B}}^{\left(t-1\right)}A\right)}{\text{tr}\left(A^{T}{\tilde{Q}}^{\left(t-1\right)}A\right)}, \end{array}$$

which is a trace ratio optimization problem has been extensively studied in [37–39]. In this paper, we use the algorithm proposed in [37] to solve the optimization problem (20). The whole procedure of FMGDA is summarized in Algorithm 1.

## 5. Experiments

In this section, we will use our FMGDA to carry out the epilepsy detection task based on the EEG data. To evaluate the performance of our FMGDA, four other Grassmann manifold algorithms are introduced, and the classification accuracy comparison results are presented.

Each comparison experiments are run for 5 rounds; 10%, 20%, 40%, 60%, and 80% of all the data are used as the training data, respectively; each round are repeated for 10 times; the average accuracy results are reported.

### 5.1. Experimental Setup

The dataset we used to evaluate the algorithm are publicly available on the web from the University of Bonn, Germany (http://www.meb.unibonn.de/epileptologie/science/physik/eegdata.html).

The complete data archive contains five groups of data (denoted by groups A to E), each group containing 100 single-channel EEG segments of 23.6 duration. The sampling rate of all data is 173.6 Hz. Groups A and B consist of segments acquired from surface EEG recording performed on five healthy volunteer subjects using standardized electrode placement scheme.

Recording was carried out when the subjects were relaxed in awaken state with eyes open (group A) and eyes closed (group B), respectively. Group C, group D, and group E are obtained from volunteer subjects with epilepsy. EEG signals in group C were recorded from the hippocampal formation of the opposite hemisphere of brain, while those in group D were measured during seizure-free intervals. Group E contains EEG signals recorded during seizure activity. In Table 1, we give a brief description of the five data sets.

**Figure 1:**
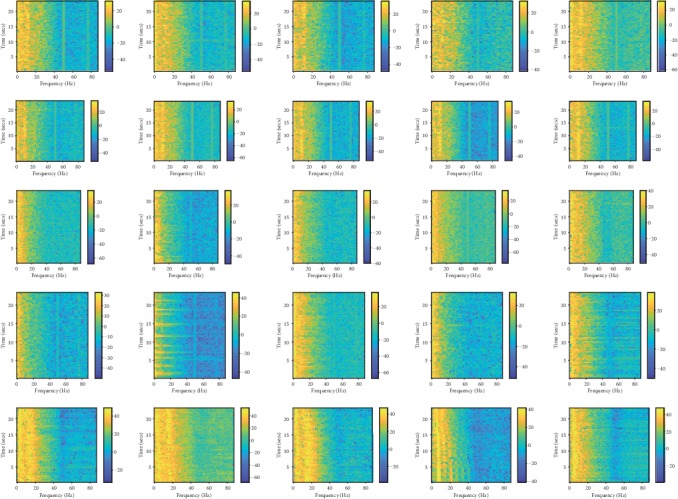
The energy distribution in the time-frequency plane feature of all EEG dataset.

In Figure 1, we present the energy distribution in the time-frequency plane of the data samples from the five group data. Five rows correspond to five different groups; in each row, there are five images which are computed by the same group EEG data. All the images are called energy distribution in the time-frequency plane. In each image, the vertical axis denotes the time, the horizontal axis represents the normalized frequency, and the color pixel denotes the energy value. The warmer color means the high energy value, and the colder color means the lower energy. The energy distribution in the time-frequency planes is computed by the short-time Fourier transform algorithm and the logarithmic operation then used to extract the logarithmic spectrogram value. All the images corresponding to the EEG data epochs are randomly chosen from the groups. Having obtained the power spectral density of EEG data, we then employ the logarithm to the spectrum power to get the logarithm feature. Finally, we process these logarithm power spectral density feature matrices as the same as the image set or video clip data.

We adopt the singular value decomposition (SVD) to get the basis of the matrix which consists of raw features of EEG spectrum set. More precisely, let {**X**_*i*_}_*i*=1_^*M*^  be a spectrum set, where **X**_*i*_ is a spectrum of EEG data with dimensionality *m* × *n* and *M* is the number of spectrum sets. By vectorizing all the spectrum and stacking them along the column, we get a matrix **Y** = [vec(**X**_1_), vec(**X**_2_), ⋯, vec(**X**_*M*_)] which can be factorized as **Y** = **UΣV**^*T*^ via SVD. We then choose the first *p* columns of **U** ∈ *𝒢*(*p*, *m* × *n*) as the Grassmannian point to represent the image set {**X**_*i*_}_*i*=1_^*M*^.

To validate the performance of our proposed FMGDA, five other Grassmann manifold algorithms are introduced into our designed experiments to make comparisons with our algorithm on the clustering performance. The competitor algorithms used in the designed experiments are listed as follows:

- KNN: *k* nearest neighbor classifier using projection metric for classifying Grassmann points without dimensionality reduction
- PML [28]: projection metric learning on the Grassmann manifold which can be seen as extension of Fisher LDA-like framework on the Grassmann manifold
- GGDA [27]: graph embedding discriminant analysis on Grassmannian manifolds
- GLPP [21]: locality preserving projections for Grassmann manifold
- FMGDA: the proposed algorithm in this paper

We first use PML, GGDA, GLPP, and FMGDA to project the high-dimensional Grassmann points to a lower-dimensional manifold, then the KNN with *k* = 1 is employed to classify Grassmann points.

### 5.2. Experiment Results and Discussion

From Figure 1, we can see the spectrum energy distribution of all the groups EEG data. Subject A data is different significantly with subject B data. We can conclude that eyes closed or open have obviously influence on the EEG data spectrum which can degrade the algorithm's performance on the epilepsy detection. Further, the intrinsic lower signal-to-noise ratio also results in the spectrum energy value to vary severely.

In the first designed experiment, we implement the five category classification task. Training data are randomly chosen from the total instances of each group data and the rest for test. We demonstrate the clustering accuracy results of the algorithms in the case of different numbers of training samples and data dimensionality in Table 2. The variable ratio denotes the percentage of all data we used for the model training, and the variable dim denotes the reduced dimensionality. From the experimental result, we can see that when the reduction dimensionality value is between 20 and 35, FMGDA has the best performance. As the dimensionality reduces and the performance decreases, the strange phenomenon also exists in other competitor algorithms. Classifying the EEG data into five different categories with only one channel data, our proposed FMGDA can achieve 70% accuracy when the setting value of the ratio and the reduction dimensions are 0.8 and 25, respectively. When the reduced dimensionality is between 20 and 35, we can get the considerable performance. The classification performance decreases as the dim increases; when the value of dim is bigger than 30, we guess that it is because too high dimensionality will introduce too much redundant information, thus degrading the algorithm's performance.

In the second designed experiment, we implement the binary classification experiment. We classify those healthy persons who are free in epilepsy with eyes open and closed. The experiment results are displayed in Table 3. From Table 3, we can get that when the dim value is equal to 15, we can get 70% classification accuracy which is significantly higher than the random probability. From this experiment, we can conclude that the state of the eyes has a very important influence on the classification of EEG signals.

In the third designed experiment, we compare the classification performance of our FMGDA with the competitor algorithms. We compare the five class classification and binary classification accuracy results, respectively. We firstly cluster the EEG data into five classes to try to recognize those healthy persons whose eyes are open and those healthy persons whose eyes are closed, those persons who get the epilepsy in the hippocampal formation of the opposite hemisphere of the brain during seizure-free intervals, within the epileptogenic zone during seizure-free intervals and those persons who are during seizure. Secondly, we perform the binary clustering task to classify the EEG data into two classes corresponding to the healthy person with eyes open and the healthy person with eyes closed.

**Figure 2:**
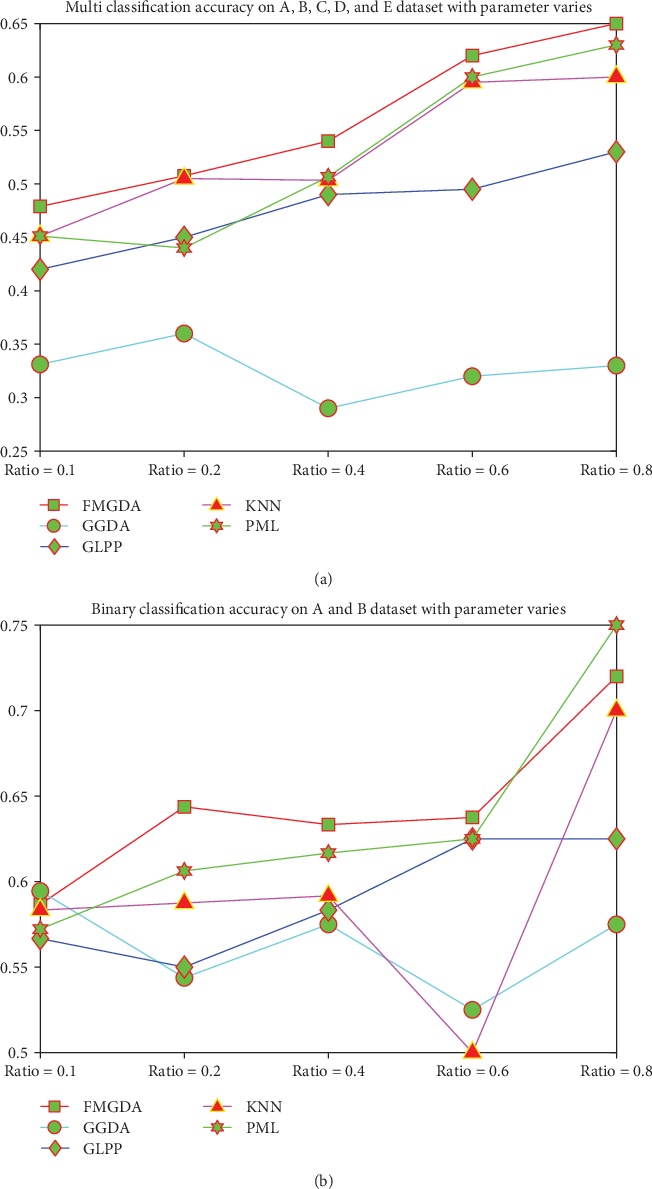
The classification accuracy on the EEG dataset with the parameter value varies. (a) Multiclass classification results over the all EEG dataset. (b) The binary classification results over group A and group B datasets.

The comparison results are displayed in Figure 2. Classification accuracy shows that our FMGDA defeats the second place algorithm by a small margin. Even in the case of some parameter settings, the performance of our algorithm is weaker than that of the KNN or PML algorithms. The binary classification performance comparisons show that eyes open or closed have a significant effect on person's EEG signal, that is to say the state of the eyes has bad influence on the detection of epilepsy based on the EEG signal. The five category classification results demonstrate that none of the algorithms can get the classification accuracy greater than 75%. We guess that on the one hand, it is due to the complexity of the human brain and the low signal-to-noise ratio of the EEG signal itself, and on the other hand, it shows that our algorithm needs to be improved.

**Figure 3:**
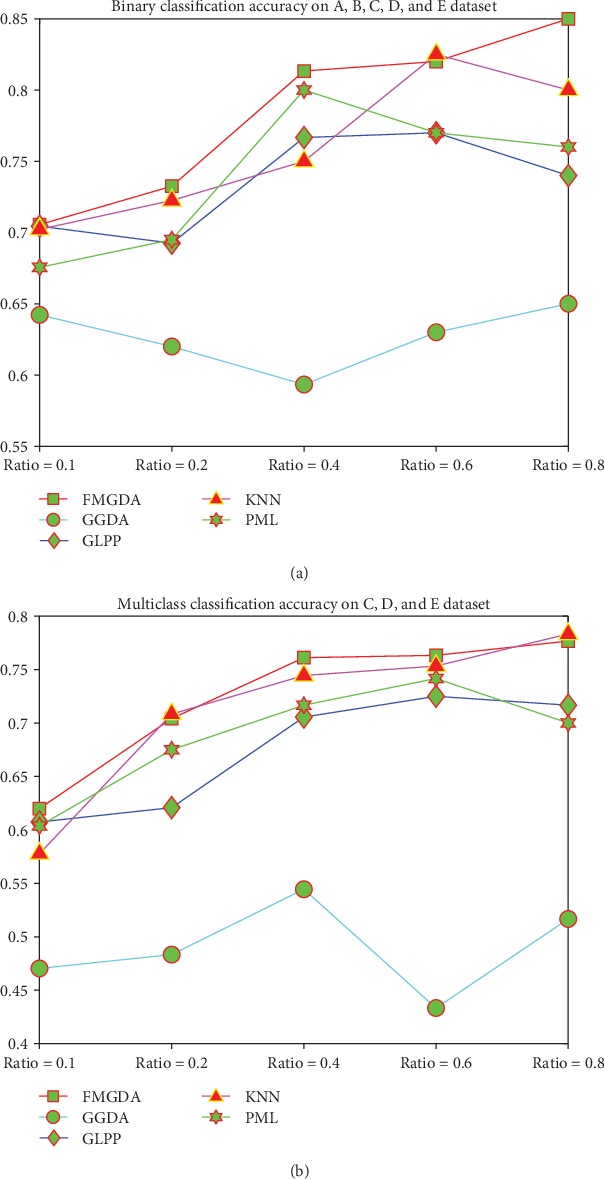
The classification accuracy on the EEG dataset with the parameter value varies. (a) Binary classification results over the all EEG dataset. (b) Multiclass classification results over group C, group D, and group E datasets.

We designed a fourth experiment to compare the performance of epilepsy detection based on Grassmann algorithms. We implement the binary classification task on all of the EEG data to classify the epilepsy persons from the healthy persons. We compare the clustering performance of our FMGDA with the competitor algorithms. The comparison results are displayed in Figure 3. From the comparison results, we can get that our proposed FMGDA algorithm shows better performance on most of the experimental parameter setting case. The KNN and PML algorithms also get good clustering results and GGDA get the worst results.

**Figure 4:**
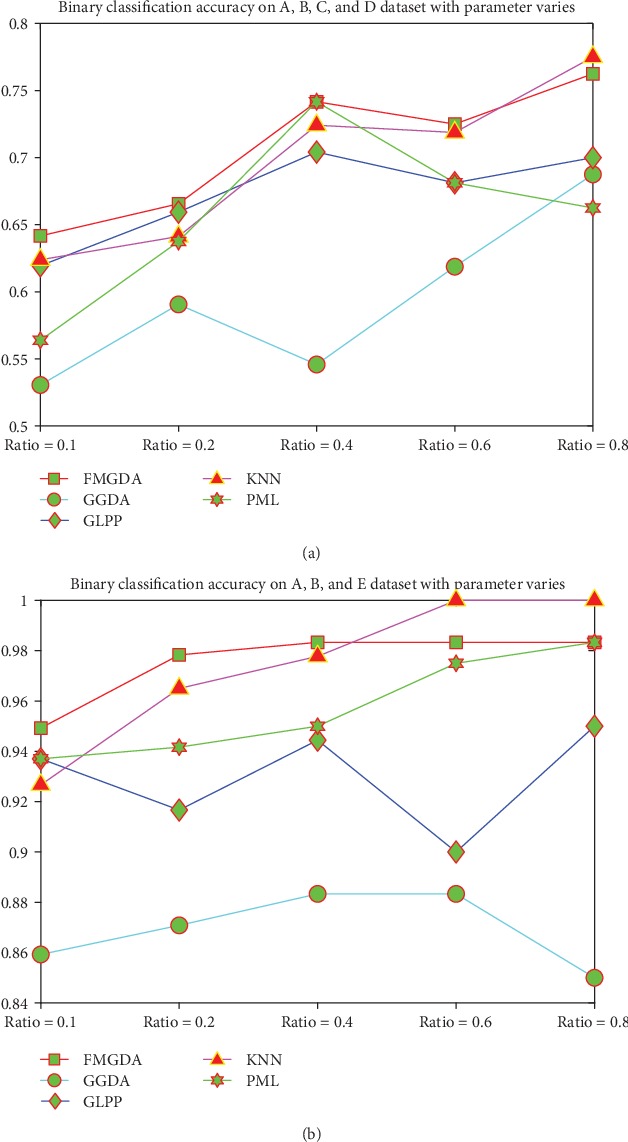
The classification accuracy on the EEG dataset with the parameter value varies. (a) Binary classification results over group A, group B, group C, and group D EEG dataset. (b) Binary classification results over group A, group B, and group E datasets.

In the fifth experiment, we test the algorithms' clustering performance of binary classification on the healthy and epilepsy data which from the healthy persons with eyes open, from healthy persons with eyes closed, and from those persons who are during seizure, the classification accuracy is presented in Figure 4. From the experimental results, we can learn that KNN algorithm has the best performance over the epilepsy detection task; our proposed FMGDA is inferior to KNN algorithm by a slight margin in some experiment cases but still better than the other three algorithms. The results also show that based on single-channel EEG data, patients, during seizures, can be detected with high accuracy and also those epileptic patients during seizure-free intervals.

## 6. Conclusion

In this paper, we proposed to use a Fréchet mean-based discriminant analysis method to implement the epilepsy detection based on the EEG data. Firstly, the short-time Fourier transform is used to extract the spectrogram feature of EEG data to form a 2D power spectrum image. Secondly, we project the high-dimensional Grassmann spectrum image to a low-dimensional Grassmann manifolds. Thirdly, we adopt the Fréchet mean method to characterize the center of Grassmann points. Finally, the classical KNN method is adopted to detect the epilepsy based on the low-dimensional Grassmann points. Experimental results on benchmark datasets have demonstrated that our proposed method is useful in the application of epilepsy detection based on the EEG data and our proposed algorithm has certain advantages over the other competitor algorithms. However, there are still existing problems in our proposed algorithm. Firstly, the performance of our FMGDA on the epilepsy detection based on the EEG signal does not have great advantages, some state-of-art algorithms have better performance than our FMGDA. Secondly, the optimization algorithm involves the single value decomposition operations, which increase the complexity of our FMGDA; when the data size increases, the algorithm will be limited. We will focus on the enhance performance as well as reduce the optimization algorithm's complexity in our future work.

## Figures and Tables

**Algorithm 1:**
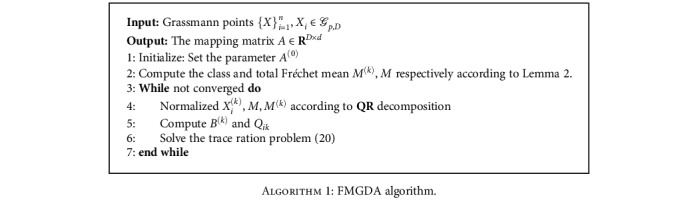
FMGDA algorithm.

**Table 1:** Dataset description.

Subject	Groups	Size	Dataset description
Healthy	A	100	EEG signals from healthy people with eyes open
	B	100	EEG signals from healthy people with eyes closed

Epileptic	C	100	EEG signals obtained in the hippocampal formation of the opposite hemisphere of the brain during seizure-free intervals
	D	100	EEG signals obtained from within the epileptogenic zone during seizure-free intervals
	E	100	EEG signals measured during seizure

**Table 2:** The performance of FMGDA algorithms in the epilepsy detection of five categories.

Ratio	dim = 4	dim = 6	dim = 8	dim = 10	dim = 15	dim = 20	dim = 25	dim = 30	dim = 35	dim = 40	dim = 45	dim = 50	dim = 55	dim = 60
0.1	0.3800	0.3822	0.3689	0.4667	0.4489	0.4156	0.4289	0.3756	0.4022	0.3867	0.3733	0.3356	0.2622	0.2267
0.2	0.4050	0.3850	0.3725	0.4250	0.4525	0.4450	0.4575	0.4275	0.3950	0.4000	0.3750	0.3325	0.3175	0.2475
0.4	0.4233	0.3600	0.4367	0.4600	0.4733	0.4933	0.5400	0.4876	0.4500	0.4667	0.4200	0.3700	0.3100	0.2433
0.6	0.5150	0.3750	0.4000	0.4900	0.5000	0.4750	0.6200	0.5600	0.4650	0.4500	0.4100	0.3800	0.2600	0.2700
0.8	0.4600	0.4100	0.5000	0.5700	0.5300	0.5800	0.5900	0.5000	0.5200	0.4750	0.4300	0.3600	0.3300	0.2200

**Table 3:** The performance of FMGDA algorithms in the epilepsy detection of two categories. All the data were classified into two categories corresponding to the healthy person and epileptic person, respectively.

Ratio	dim = 4	dim = 6	dim = 8	dim = 10	dim = 15	dim = 20	dim = 25	dim = 30	dim = 35	dim = 40	dim = 45	dim = 50	dim = 55	dim = 60
0.1	0.6722	0.5500	0.6444	0.5833	0.5657	0.5444	0.5556	0.5778	0.5667	0.4833	0.5722	0.5167	0.4889	0.4833
0.2	0.7750	0.5625	0.6313	0.6250	0.64338	0.5938	0.6063	0.5438	0.5125	0.5250	0.5750	0.5188	0.5375	0.4688
0.4	0.5583	0.5833	0.6500	0.6583	0.5333	0.5833	0.5917	0.5833	0.5250	0.5750	0.6083	0.4333	0.5167	0.5667
0.6	0.6000	0.6125	0.6125	0.5500	0.6375	0.5375	0.6375	0.6625	0.5875	0.4125	0.4875	0.4750	0.5500	0.5250
0.8	0.6000	0.5000	0.6000	0.6750	0.7000	0.6250	0.6500	0.6250	0.6000	0.6250	0.3250	0.5250	0.5500	0.6500

## Data Availability

Data were used to support this study are public dataset, which are available C at:http://www.meb.unibonn.de/epileptologie/science/physik/eegdata.html.
